# Immunological and Nonimmunological Effects of Indoleamine 2,3-Dioxygenase on Breast Tumor Growth and Spontaneous Metastasis Formation

**DOI:** 10.1155/2012/173029

**Published:** 2012-05-13

**Authors:** Vera Levina, Yunyun Su, Elieser Gorelik

**Affiliations:** ^1^Department of Medicine, University of Pittsburgh Cancer Institute, Pittsburgh, PA 15213, USA; ^2^University of Pittsburgh Cancer Institute, Hillman Cancer Center, Pittsburgh, PA 15213, USA; ^3^Department of Immunology, University of Pittsburgh Cancer Institute, Pittsburgh, PA 15213, USA; ^4^Department of Pathology, University of Pittsburgh Cancer Institute, Pittsburgh, PA 15213, USA

## Abstract

The role of the tryptophan-catabolizing enzyme, indoleamine 2,3-dioxygenase (IDO1), in tumor escape and metastasis formation was analyzed using two pairs of *Ido1+* and *Ido1−* murine breast cancer cell lines. *Ido1* expression in 4T1 cells was knocked down by shRNA, and *Ido1* expression in NT-5 cells was upregulated by stable transfection. Growth of *Ido1−* tumors and spontaneous metastasis formation were inhibited in immunocompetent mice. A higher level of cytotoxic T lymphocytes was generated by spleen cells from mice bearing *Ido1−* tumors than *Ido1+* tumors. Tumor and metastatic growth was enhanced in immunodeficient mice, confirming an intensified immune response in the absence of *Ido1* expression. However, *Ido1+* tumors grow faster than *Ido1−* tumors in immunodeficient SCID/beige mice (lacking T, B, and NK cells) suggesting that some *Ido1*-controlled nonimmunological mechanisms may be involved in tumor cell growth regulation. *In vitro* experiments demonstrated that downregulation of *Ido1* in tumor cells was associated with decreased cell proliferation, increased apoptosis, and changed expression of cell cycle regulatory genes, whereas upregulation of *Ido1* in the cells had the opposite effects. Taken together, our findings indicate that *Ido1* expression could exert immunological and nonimmunological effects in murine breast tumor cells.

## 1. Introduction

Immune escape is one of the hallmarks of cancer [1]. There are a variety of active mechanisms of immune suppression that are elaborated by tumors to drive immune escape, such as the loss of MHC class I molecules or tumor antigens, induction of T regulatory cells, and the production of various immunosuppressive molecules (IL-10, TGF-*β*, adenosine, PGE2, etc.) [2–4]. One mechanism of immune escape that has been linked to cancer is elevation of the tryptophan-catabolizing enzyme, indoleamine 2,3-dioxygenase (IDO1) [5–14].

IDO1 can be expressed in many tissue and cell types, such as placenta, lung, gut, and epididymis [13, 15–17]. The expression of IDO1 at the maternal-fetal interface in mice placenta is believed to play a role in the protection of the fetus from immunologic rejection by maternal immune mechanisms [18]. Indeed, this has been supported experimentally wherein the pharmacological inhibition of IDO1 by 1-methyltryptophan (1MT) resulted in the rejection of allogeneic, but not syngeneic, murine fetuses [19–21]. *Ido1* activation occurs commonly in tumor cells and/or tumor-draining lymph nodes (TDLNs), and pharmacological inhibition of IDO1 with 1-MT has been shown to result in T-cell-dependent antitumor responses in animal models [8, 22–27]. 1-MT was observed to retard tumor outgrowth but was unable to trigger complete tumor regression [6]. *In vitro* studies indicate that IDO1 is capable of exerting suppressive effects directly on T cells and can activate suppressive populations of regulatory T cells [8, 9]. Furthermore, soluble cytotoxic-T-lymphocyte-antigen-4- (CTLA4-) expressing T regulatory cells induce IDO1 expression in DC, converting them into regulatory antigen-presenting cells (APCs) [24, 26]. Intracellular signaling via CD80/86, CD200R, and Fc*ε*RI could induce IDO1 expression by DC [9, 13, 25]. A tumor-related immune escape mechanism based on tryptophan degradation by IDO1 has been proposed [22, 28]. *Ido1* is expressed by tumor cells; however, the level of *Ido1* expression is significantly lower than in placenta. Tumor cell inhibition of immune response was only demonstrated for *Ido1*-transfected clones, exhibiting 1000-fold increased expression of *Ido1* mRNA relative to placental levels [22, 29]. Thus, a role for IDO1 in tumor immune response is indicated but requires further investigation.

In this study, we examined the impact of IDO1 on tumor growth and metastasis in immune-competent and immune-deficient mice. Two murine breast cell lines, 4T1 and NT-5, expressing *Ido1* and missing *Ido1* expression, respectively, were utilized. NT-5 cells were transfected with an *Ido1* expression vector to establish an NT-5/*Ido1+* line. Expression of *Ido1* in 4T1 cells was knocked down by transfection with an *Ido1−* specific shRNA expressing plasmid to establish a 4T1/*Ido1−* line. Using these two pairs of cell lines, we examined the relationship between *Ido1* expression and cancer cell growth *in vitro* and *in vivo.* Our analysis of tumor growth and metastasis, in immunocompetent and immunodeficient mice, revealed that IDO1 not only modulated the immunological system, but also played an important biological role in tumor cell proliferation, cell cycle regulation, and antiapoptotic signaling.

## 2. Materials and Methods

### 2.1. Tumor Cell Lines

The NT-5 HER-2/neu-expressing tumor cell line was provided by Elizabeth Jaffe, John Hopkins University. The 4T1 mouse mammary tumor cell line was purchased from American Type Culture Collection. Cells were cultured in RPMI-1640 medium (Cellgro Mediatech, Inc, Manassas, VA) supplemented with 10% FBS (Sigma-Aldrich Co, St. Luis, MO).

### 2.2. Plasmid Construction and Cell Transfection

The mammalian expression vector for *Ido1* was constructed by inserting full-size mouse *Ido1* cDNA into the vector pRc/CMV (Invitrogen, Life Technologies Corp., Carlsbad, CA). NT-5 cells were cloned, and IDO expression in the individual clones was evaluated. The clone with the lowest IDO1 expression was used for transfection with either *Ido1* constructs or control pRc/CMV vector using Lipofectamine 2000 according to manufacturer instructions (Invitrogen). Stable transfectants (NT-5/*Ido1+* and NT-5/vector) were selected by growth in a medium supplemented with 400 *μ*g/mL G418 (Sigma-Aldrich Co). IDO-positive 4T1 breast tumor cells were also cloned, and the clone with highest IDO expression was transfected with shRNA against IDO containing plasmid and the control plasmid (SABiosences). Stably transfected 4T1/*Ido1−* and 4T1/vector cells were selected with 600 *μ*g/mL of G418 (Sigma-Aldrich).

### 2.3. Real-Time PCR and Gene Expression Profiling

RNA was isolated from the cells using the RNeasy kit from Qiagen (Valencia, CA), and the extracted RNA was converted to cDNA using the RT^2^ First Strand Kit from Super Array Bioscience Corporation (Frederick, MD) according to manufacturer's protocols. Real-time PCR was carried out using the Super Array RT^2^ Real-Time SYBR Green PCR Master Mix (Super Array Bioscience) and was performed on the ABI Prism 7700 sequence detector real-time PCR system (AB Applied Biosystems, Foster City, CA). Primers for mouse *Ido1* were forward 5′-GTACATCACCATGGCGTATG-3′; reverse: 5′-CGAGGAAGAAGCCCTTGTC-3′. Standard curves were generated from five 10-fold serial dilutions of tumor cell cDNA, and no product could be observed in the negative control lacking template. Differences in gene expression were calculated by using the ∆Ct method and normalized to GAPDH according to the manual from Super Array Bioscience (Super Array, Bioscience Corp., Frederick, MD).

The RT^2^ Profiler PCR Array System and mouse cell cycle regulation RT^2^ Profiler PCR Array (Super Array, Bioscience Corp) were used. Real-time PCR detection was carried out per the manufacturer's instructions. The experimental cocktail was prepared by mixing cDNA isolated from cell lines with the RT^2^ Real-Time SyBR Green/ROX qPCR Master Mix (SABiosciences Corp., Frederick, MD). The mixtures were equally aliquoted into the 96-well plate containing predispensed gene-specific primer sets, then real-time PCR was performed using the ABI Prism 7900HT (ABI, Applied Biosytems Corp., Foster City, CA). Differences in genes expression were compared between 4T1/vector and 4T1/*Ido1−* cells or between NT-5/vector and NT-5/*Ido1+* cells. Analyses of the raw data were done through the Super Array Data Analysis Web Portal (Super Array Bioscience Corp.).

### 2.4. Enzymatic Assay for IDO Activity

IDO activity was determined using what is described in [10, 30]. Briefly, cell extracts were prepared, mixed with an equal volume of 2x reaction buffer (100 mM PBS, 40 mM ascorbate, 20 *μ*M methylene blue, 200 *μ*g/mL catalase, 800 *μ*M L-Tryptophan (Affymetrix, USB Inc, Cleveland, OH), pH 6.5), and incubated at 37°C for 30 min in order to permit IDO-mediated conversion of L-Trp into N-formylkynurenine. The reaction was stopped with 30% trichloroacetic acid, and samples were incubated at 50°C for 30 min to hydrolyze the N-formylkynurenine produced to kynurenine. After centrifugation, supernatants were collected and mixed with Ehrlich's reagent (0.8% p-dimethylaminobenzaldehyde in acetic acid) in 96-well plates, and absorbance at 490 nm was measured on a microplate reader. IDO activity is defined as the amount of enzyme required to produce 1 nmol of kynurenine per hr per mg of protein.

### 2.5. *In Vitro* Cell Proliferation Assay

The cells were cultured in a 96-well plate for 2 days and pulsed with 0.2 *μ*Ci/well of ^3^H-thymidine overnight followed by collection of supernatant. 50 uL of supernatant was mixed with 2 mL of CytoSpin (MP, Biomedicals LLC, Solon, OH) and counted by liquid scintillation counter (Wallac). The tumor cells were also counted by Cellomics Array Scan HCS Reader (Thermo Fisher, Pittsburgh, PA) following 3 days in culture as described previously [31].

### 2.6. Flow Cytometry Analysis

Cells were cultured in a control medium or in serum starvation conditions (RPMI with 0.1% FBS or 0.3% FBS) for 3 days followed by culture in RPMI with 10.0% FBS for 24 hours. Cells were stained with FITC-conjugated antibromodeoxyuridine (BrdU) and/or propidium iodide (PI) using FITC BrdU Flow Kit (BD Biosciences, Pharmingen, San Diego, CA) according to the manufacturer's instruction. Flow cytometry was performed by CYAN ADP (Beckman Counter, Brea, CA). The data were analyzed with the Cytomation Summit v4.3 software.

### 2.7. *In Vivo* Local and Metastatic Tumors Assays

BALB/c, FVB/N, and SCID-beige were purchased from The Jackson Laboratory (Bar Harbor, ME) and used at 8-9 weeks of age. Mice were housed in the University of Pittsburgh Cancer Institute animal facility which is accredited for animal care by the American Association of Laboratory Animal Care. Experiments were performed in accordance with the approved institutional protocol and the guidelines of the Institutional Animal Care and Use Committee. BALB/c and SCID-beige mice were inoculated s.c. with 2.5 × 10^4^ or 1.0 × 10^5^ of 4T1/vector cells or 4T1/*Ido1−* cells per mouse; FBV/N and SCID-beige mice were inoculated s.c. with 5 × 10^6^ of NT-5 or NT-5/*Ido1+* cells per mouse. Tumor growth was evaluated by measurement of tumor diameters 3 times a week, and the tumor volume was calculated as length × width^2^ × 0.52. All data is represented as mean ± SE. Experiments were terminated when tumors reached 2.0 cm in diameter. Each group contained 10 mice. Experiments were repeated twice. Lungs were harvested when tumors reached approximately 2 cm in diameter and fixed in Bounce solution. Metastases were counted under a dissecting microscope.

### 2.8. Cytotoxicity Assay

Spleen cells from BALB/c mice bearing 4T1/*Ido1−* or 4T1 tumors were cultured with irradiated (15,000 rad) 4T1/*Ido1−* or 4T1 tumor cells for 5–8 days in the presence of 300 IU/mL of IL-2 in 24-well plates at the ratio as 3 × 10^6^ spleen cells: 1 × 10^6^ tumor cells. The cytotoxic activity of restimulated spleen cells was tested against ^51^Cr-labeled 4T1/*Ido1−* or 4T1 cells at the effector: target ratio as 100 : 1. Spleen cells were distributed into V-96-well plates preloaded with ^51^Cr-labeled tumor cells; 96-well plates containing cell mixtures were centrifuged at 2000 rpm for 2 minutes and then incubated 4 hours at 37°C. 25 *μ*L of supernatant was transferred into yttrium silicate scintillator-coated microplates (LumaPlate-96, PerkinElmer) and left overnight at RT. The level of *β*-emission released by ^51^Cr was measured in a *β*-counter.

### 2.9. Western Blot Analysis

The cells were lysed in RIPA buffer supplemented with 1% CLAP cocktail (antipan, leupeptin, pepstatin, and chymostatin) and 1 mM PMSF. 50 ug of protein extracts were resolved on 4.5–12% of SDS-PAGE and transferred to PVDF membranes (Bio-Rad, Hercules, CA). The membranes were blocked with 5% nonfat dry milk in TBST buffer, probed with antibodies against stratifin (1 : 200, Sigma), and Atm (1 : 200, Santa Cruz Biotechnology, Inc, Santa Cruz, CA) for two hours at RT or overnight at 4°C, and then incubated with horseradish peroxidase-labeled secondary antibody (Santa Cruz). The signals were detected by ECL (Amersham). Films were scanned and analyzed by Image-QuanT data analysis software (Molecular Dynamics).

### 2.10. Statistical Analysis

Data are presented as mean ± SD. Comparisons between values were performed using a two-tailed Student's *t*-test. For the comparison of multiple groups, a one- or two-way ANOVA test was applied. For all statistical analyses, the level of significance was set at a probability of *P* < 0.05. All experiments were repeated 2-3 times.

## 3. Results

### 3.1. Establishment of Two-Paired Stable Clones (*Ido1−* and *Ido1+*) of Murine Breast Cancer Cells

Initially, we measured *Ido1* expression in a panel of mouse tumor cell lines of differing histological origin. Two mouse breast tumor cell lines, 4T1 and NT-5, which showed differential expression of *Ido1*, were chosen. 4T1 cells, derived from sporadic breast tumor in BALB/c mice, are highly aggressive, metastatic, and poorly immunogenic [32, 33]. NT-5 breast tumor cells are derived from HER-2/*neu*-transgenic FBV/N mice, and they are immunogenic [34].

We found that 4T1 cells express *Ido1*, whereas the level of *Ido1 *gene expression in NT-5 cells was very low (*Ido1−*). Clonal variation could be attributable whenever transfection is performed on a polyclonal whole cell population. To avoid this, our transfections were designed as follows: 4T1 breast tumor cells were cloned using a standard approach for selecting single-cell clones. *Ido1* expression, in these individual cell clones, was evaluated. The clone the of the 4T1 tumor cells with the highest *Ido1* expression was transfected with *Ido1* siRNA and a control vector to generate stable 4T1/*Ido1−* and 4T1/vector clones.

Similarly, NT-5 cells (expressing *Ido1* at a very low level) were also cloned, and the clone of NT-5 cells, with the lowest level of *Ido1* expression, was selected for transfection with cDNA encoding *Ido1* or control plasmid.

**Figure 1:**
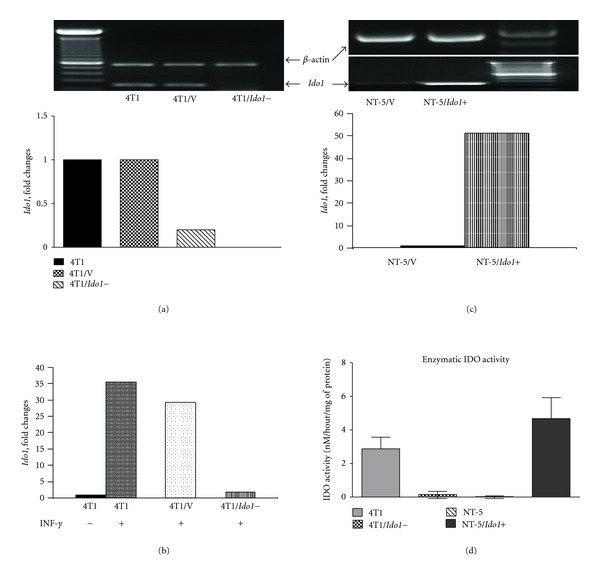
Generation of two pairs of* Ido1+* and *Ido1−* clones from murine breast cancer cells. (a) RT-PCR (top picture) real-time PCR (bottom picture) analysis of *Ido1 *expression in 4T1 cells, cells transfected with vector (4T1/V), and cells transfected with shRNA for *Ido1* (4T1/*Ido1−*). Fold change was compared with 4T1. (b) Real-time PCR analysis of the effect of INF-*γ* treatment (INF-*γ*, 25 ng/mL, 24 hours) on *Ido1 *expression in 4T1, 4T1/V, and 4T1/*Ido1− *cells. (c) *Ido1 *expression in NT-5 cells transfected with vector (NT-5/V) and cells transfected with *Ido1 *cDNA (NT-5/*Ido1+*) detected by RT-PCR (top picture) and by Real-time PCR (bottom picture). (d) Enzymatic IDO activity in 4T1, 4T1/*Ido1−*, NT-5, and NT-5/*Ido1+* cells. IDO activity is defined as the amount of enzyme required to produce 1 nmol of kynurenine per hr per 1 mg of protein. All values are the means of four measurements.

As shown in Figure 1(a), very low *Ido1* expression was detected by RT-PCR in 4T1/*Ido1−* cells compared with 4T1 and 4T1/vector cells. The quantitative RT-PCR analysis revealed 78% downregulation of *Ido1* in 4T1/*Ido1−* cells, and no changes of *Ido1* expression in 4T1/vector cells in comparison with naïve 4T1 cells (Figure 1(a)).

IFN-*γ* is a known inducer of *Ido1* [30]. We tested whether *Ido1* expression could be induced by IFN-*γ* in 4T1, 4T1/vector, and 4T1/*Ido1−* cells. *Ido1* expression was strongly increased (30-fold change) in 4T1, and 4T1/vector cells were treated with IFN-*γ* (Figure 1(b)). However, *Ido1* activation was abrogated in shRNA-transfected cells (4T1/*Ido1−*) (Figure 1(b)).

We transfected an *Ido1 *vector and scramble vector into the *Ido1 *low-expressing NT-5 cells and established stable *Ido1* expressing NT-5/*Ido1+* and NT-5/vector clones. RT-PCR analysis showed *Ido1* expression in NT-5/*Ido1+* cells, and very low expression in NT-5/vector cells (Figure 1(c) top); quantitative RT-PCR demonstrated a 50.9-fold increase of *Ido1* expression in NT-5/*Ido1+* cells in comparison to NT-5/vector cells (Figure 1(c)).

Next, we tested enzymatic activity of IDO in 4T1, 4T1/*Ido1−* NT-5, and NT-5/*Ido1+* cells (Figure 1(d)). 4T1/*Ido1−* cells demonstrated a significant reduction of IDO activity in comparison with 4T1 cells, while NT-5/*Ido1+* cells showed a significant increase in IDO activity when compared with NT-5 cells (Figure 1(d)).

### 3.2. Effect of *Ido1* Expression on Tumor Growth and Metastasis Formation

**Figure 2:**
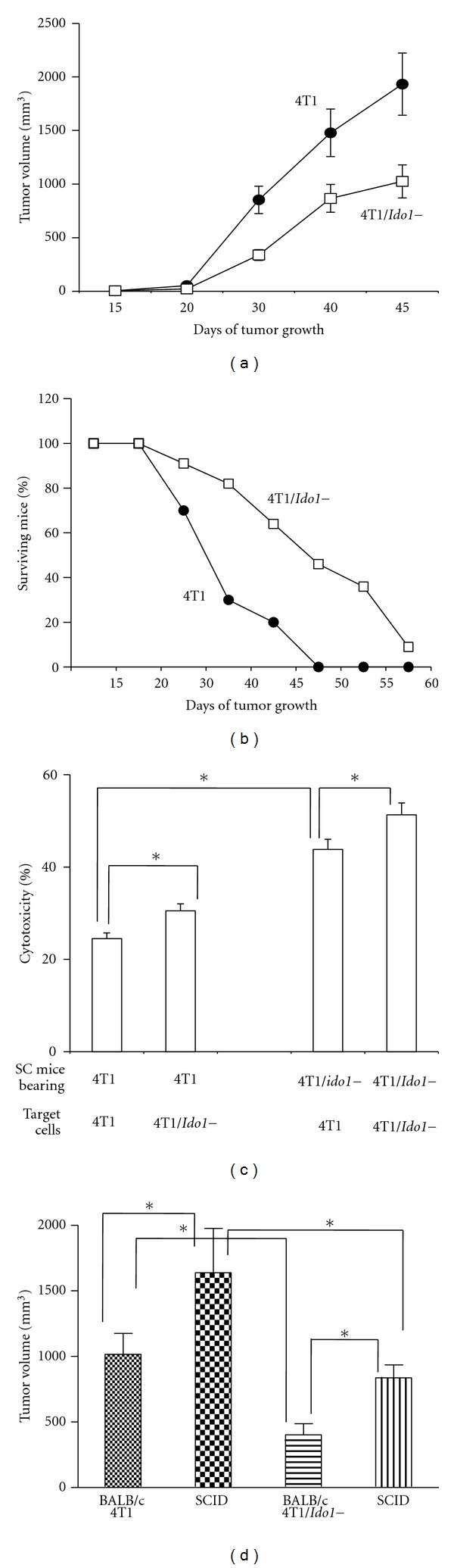
Effect of *Ido1* expression on tumor growth and spontaneous metastasis formation. (a) BALB/c mice were inoculated s.c. with 1 × 10^5^cells/mouse of 4T1/vector or 4T1/*Ido1−* tumor cells, and tumor growth was monitored. (b) Percentage of surviving mice bearing 4T1/vector and 4T1/*Ido1−* tumors. (c) Cytotoxic activity of spleen cells from mice bearing 4T1 or 4T1/*Ido1−* tumors against 4T1 and 4T1/*Ido1−* tumor cells. Spleen cells from mice bearing 4T1/vector or 4T1/*Ido1−* tumors were cultured with irradiated 4T1/vector tumor cells and 300 IU/mL of IL-2 for 5–8 days. The cytotoxic activity of the spleen cells was tested against ^51^Cr-labeled 4T1 or 4T1/*Ido1−* cells at the E : T ratio 100 : 1. (d) Growth of 4T1 and 4T1/*Ido1−* tumors in immunocompetent BALB/c and immunodeficient SCID-beige mice.

To determine the role of IDO1 in tumor growth, BALB/c mice were inoculated s.c. with 4T1/vector or 4T1/*Ido1−* tumor cells. 4T1/*Ido1−* tumors grew significantly (*P* < 0.05) slower than 4T1/vector tumors (Figure 2(a)). Mice bearing 2 cm tumors were sacrificed according to our IACUC-approved protocol, and surviving mice bearing tumors of less than 2 cm were counted at sequential time points in order to evaluate survival. At the time of complete mortality in the control group, we observed nearly 50% survival of mice bearing *Ido1−* tumors (Figure 2(b)).

The elevated survival of mice bearing 4T1/*Ido1−* tumors may be a result of a less efficient immune response against tumor cells demonstrating inhibited *Ido1* expression. To assess the immune response of BALB/c mice to 4T1/vector and 4T1/*Ido1−* tumors, spleens of mice bearing tumors were harvested, and spleen cell suspensions were prepared. Freshly prepared spleen cells had no cytotoxic activity against 4T1 tumor cells. Therefore, spleen cells were stimulated with irradiated 4T1 tumor cells for 5–8 days in the presence of IL-2 (300 IU/mL). We observed no difference in the abilities of irradiated 4T1/*Ido1−* and 4T1 (*Ido1+*) cells to stimulate spleen cells and generate cytotoxic T cells. The cytotoxic activity of stimulated spleen cells appeared after 5 days of stimulation and became more prominent after 7-8 days. Spleen cells obtained from irradiated 4T1/*Ido1 *tumor cells from 4T1/*Ido1−* tumor bearing mice demonstrated a higher cytotoxic activity than spleen cells from mice bearing 4T1/vector tumors (Figure 2(c)). Furthermore, targeted 4T1/*Ido1−* tumor cells were more sensitive to the cytotoxic activity of spleen cells than 4T1/vector cells (Figure 2(c)). Spleen cells cultured in IL-2 (300 IU/mL) without irradiated tumor cells showed no cytotoxic activity (data no shown). These results indicate that inhibition of 4T1/*Ido1−* tumor growth in BALB/c mice could be due to an elevated immune response against these cells as well as a greater sensitivity of 4T1/*Ido1−* cells to immune destruction.

To further test whether 4T1/*Ido1−* tumor growth inhibition is immunologically mediated, immunocompetent BALB/c mice or immunodeficient SCID-beige mice (which lack T, B, and NK cells) were inoculated s.c. with 4T1/vector and 4T1/*Ido1−* cells. Our expectation was that if differences in *Ido1+* and *Ido1−* tumor growth are immune modulated, then both tumors should grow similarly in SCID/beige mice. Tumor growth from *Ido1−* cells was inhibited in comparison with *Ido1+* cells in BALB/c mice. As shown in Figure 2(d), both *Ido1−* and *Ido1+* tumors grew faster in immunodeficient SCID/beige mice than in immunocompetent BALB/c mice. However, *Ido1+* tumors grew faster than *Ido1−* tumors in SCID/beige mice suggesting that some *Ido1-*controlled nonimmunological mechanisms may be involved in tumor cell growth regulation.

To test whether spontaneous pulmonary metastasis formation is dependent on *Ido1* gene expression, the number of metastases in BALB/c and SCID/beige mice was counted when tumors reached 2 cm in diameter. We found that 4T1/*Ido1−* cells generated significantly less metastasis in the lungs of BALB/c mice than 4T1 cells (92 versus 396) (Table 1). The pulmonary metastases observed in immunodeficient SCID-beige mice bearing *Ido1−* tumors were higher than in the immunocompetent BALB/c mice bearing *Ido1−* tumors (Table 1).

 To further elucidate the role of IDO1, in tumor growth and metastasis formation, we used NT-5/*Ido1−* cells and NT-5/*Ido1+* tumor cells and FVB/N mice that are immunocompetent for NT-5 cells. It is known that NT-5 breast tumor cells, derived from HER-2/*neu*-transgenic FBV/N mice, are immunogenic [34]. Indeed, tumor growth was prevented when 1 × 10^6^ NT-5 cells were inoculated into mice. However, inoculation of 5 × 10^6^ NT-5 cells resulted in tumor development. Tumor growth in many FVB/N mice was associated with enormous necrosis which made it difficult to measure tumor size; therefore, mice were sacrificed when they showed signs of moribund, and lung metastases were counted. We found that NT-5/*Ido1+* cells are more metastatic than NT-5/*Ido−*cells in immunocompetent FVB/N mice (Table 1). The numbers of metastases developed from NT-5/*Ido1+* cells and NT-5/*Ido−* cells in immunodeficient SCID/beige mice were significantly higher than in FVB/mice (Table 1). However, even in immunodeficient mice, NT-5/*Ido1−* cells developed less metastasis than NT-5/*Ido1+* cells.

 Taken together, these results indicate that *Ido1* plays role in tumor growth and metastasis formation.

### 3.3. Effect of *Ido1* Expression on Apoptosis Changes Cell Cycle and Tumor Cell Proliferation *In Vitro*

The differences in *Ido1+* and *Ido1−* tumor growth in immunodeficient mice indicate a possible role of *Ido1* in apoptosis and tumor cell proliferation. To analyze cell cycle distribution, in *Ido1+* and *Ido1−* cells, we used flow cytometry and FITC-conjugated antibromodeoxyuridine (BrdU) and/or propidium iodide (PI) staining. The approach relies on the detection of 5′-bromo-2′-deoxyuridine (BrdU) incorporation which identifies DNA-replicating cells, and PI staining reveals the distribution of cells in three major phases of the cycle (G1 versus S versus G2/M) and makes it possible to detect apoptotic cells with fractional DNA content.

**Figure 3:**
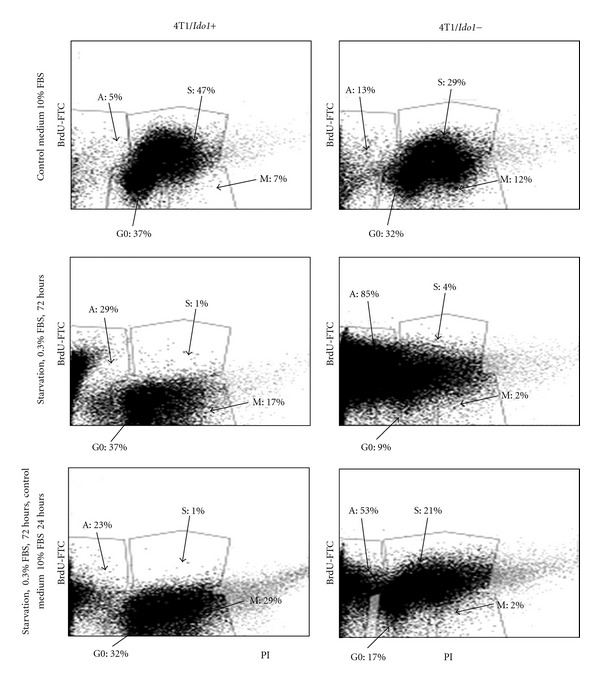
Flow cytometry analysis of apoptosis and cell cycle in *Ido1+* and *Ido1−* cells. 4T1 and 4T1/*Ido1−* cells were cultured in normal medium (10% FBS), or under starvation condition (0.3% FBS) for 3 days followed by a 24 hour recovery in normal medium. Flow cytometry analyses of cells stained with FITC-conjugated antibromodeoxyuridine (BrdU) and propidium iodide (PI) were performed. The data are presented as a percentage of cells in apoptosis (A), G0, S, and M phases.

**Figure 4:**
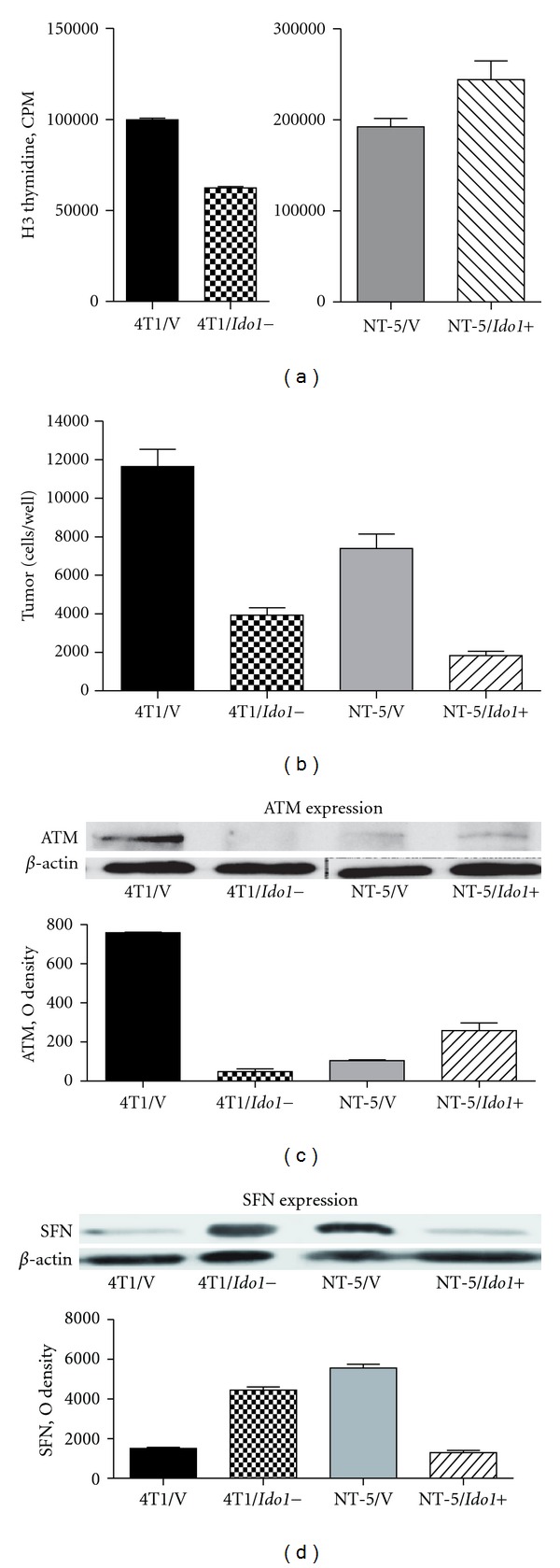
*In vitro* proliferation and western blot analysis ATM and SFN proteins in *Ido1+* and *Ido1−* breast tumor cells. (a) Analysis of ^3^H thymidine incorporation and (b) cell counting using Cellomics Array Scan in 4T1/vector, 4T1/*Ido1−, *NT-5/vector, and NT-5/*Ido1+* cells. (c) Western blots analysis of ATM and (d) SFN (14-3-3 sigma protein) expression in 4T1/vector, 4T1/*Ido1−*, NT-5/vector, and NT-5/*Ido1+* cells.

Figure 3 represents the results of the experiments in which double staining was applied. Bivariate distribution (contour map) of DNA content and BrdU incorporation in 4T1 and 4T1/*Ido1−* cells are shown. The 4T1/*Ido1−* cells growing in control media have significantly (*P* < 0.05) higher percentage of apoptotic cells than 4T1/*Ido1+* cells. Starvation is a condition that often occurs within the tumors. We analyzed the effect of starvation conditions (low serum in culture medium) on apoptosis induction in 4T1/*Ido1+* and 4T1/*Ido1−* cells. Here, the proportion of apoptotic 4T1/*Ido1−* cells reached 86%, which is approximately 3 times higher than that observed in the 4T1/*Ido1+* cell population. The 4T1/*Ido1+* cells recover faster after starvation (Figure 3). Cell cycle phase distribution was also different in 4T1/*Ido1+* and 4T1/*Ido1−* cell populations in control media and under starvation (Figure 3). Control cells showed a significantly higher proportion of cells in the S phase than 4T1/*Ido1− *cells. 4T1/*Ido1+* growing in low serum demonstrated a higher proportion of cells in M and G0 phases than 4T1/*Ido1− *cells. We then compared the proliferation of 4T1/*Ido1+* and 4T1/*Ido1−* cells as well as NT-5/*Ido1−* and NT-5/*Ido1+* cells *in vitro*. The results of ^3^H-thymidine incorporation showed a significant reduction in proliferation of *Ido1−* cells in comparison to *Ido1+ *cells (Figure 4(a)). Next, the same pairs of *Ido1+*/*Ido1−* cells were grown for 3 days and counted as described previously [31]. As shown in Figure 4(b), the inhibition of *Ido1 *activity in 4T1 cells was associated with an inhibition in 4T1/*Ido1−* cell proliferation, whereas overexpression of *Ido1* in NT5/*Ido1+* cells stimulated tumor cell proliferation.

### 3.4. Ido1 and Cell Cycle Gene Expression

To further analyze the differences of the cell cycle regulation in *Ido1+* and *Ido1−* cells, the cell cycle genes expressions were investigated (Table 2). We used the Mouse Cell Cycle PCR Array from SABiosciences to compare cell cycle gene expression in two pairs of positive and negative *Ido1* tumor cells.

*Ido1* inhibition in 4T1 tumor cells was associated with significant changes in the expression of 40 cell cycle genes involved in the G1/S transition, S phase, G2/M transition, checkpoints, and M phase, and the majority of the altered genes were downregulated. *Ido1* expression in NT-5/*Ido1+* cells correlated with changes in the expression of 14 genes, 13 of which were upregulated (Table 2). The expression of four genes (Trp63(p63), dystonin (Dst), stromal antigen1 (Stag1) and microtubule-actin crosslinking factor (Macf1)) was lower in *Ido1* downregulated cells, and higher with *Ido1 *upregulation (Table 2). To verify whether the changes in gene expression are associated with upregulation of IDO1 protein expression in *Ido1+* and *Ido1−* cells, we performed a western blot analysis of the cell cycle regulators ATM and stratifin (Sfn) [35, 36].

*Atm *(ataxia telangiectasia mutated) is an important cell cycle regulation gene encoding a serine/threonine protein kinase that is recruited and activated by DNA double-strand breaks. ATM phosphorylates several key proteins that initiate activation of the DNA damage checkpoint, leading to cell cycle arrest, DNA repair, or apoptosis [35, 36]. *Atm *expression was significantly decreased (−18.3-fold) in 4T1/*Ido1−* cells and only slightly changed (1.7-fold) in NT-5/*Ido1+* (Table 2). As demonstrated in Figure 4(c), ATM is expressed in 4T1/vector (*Ido1+*)* cells*, and ATM production is dramatically reduced in *Ido1−* cells.

Stratifin (Sfn), also named 14-3-3 sigma, is a multifunctional protein involved in cell cycle regulation [36]. Downregulation of *Ido1 *in 4T1 cells is associated with upregulation of Sfn gene expression (12 fold); and upregulation of *Ido1 *in NT5 cells is associated with a decreased level (*−*7 fold) of Sfn gene expression (Table 2).

 Western blot analysis demonstrated that SFN protein is increased in *Ido1−* cells, and it is reduced in *Ido1+* cells (Figure 4(d)). Thus, the results of the PCR analysis of *Atm* and* Sfn* gene expression are consistent with the results of the western blot analysis of the ATM and SFN proteins

## 4. Discussion

We first analyzed the impact of *Ido1* expression on tumor growth and spontaneous pulmonary metastasis formation and then investigated the IDO1-regulated mechanisms involved in tumorigenesis. One of the key experimental strategies to elucidate the function of a gene *in vitro* and *in vivo* is the specific inhibition or upregulation of its expression. Here, we explored both approaches. *Ido1+* cells were transected with plasmid expressing shRNA for *Ido1*, and *Ido1− *cells were transfected with *Ido1* cDNA. These two pairs of *Ido+ *and* Ido− *clones were evaluated throughout our investigation.

We found that 4T1/*Ido1+* tumors grew faster than 4T1/*Ido1−* tumors in immunocompetent mice. Growth inhibition of *Ido1−* tumor cells can be attributed to a more efficient immune response. This conclusion is based on the following. (1) Spleen cells from mice bearing 4T1/*Ido1−* tumors generated a higher level of cytotoxic T cells (CTCs). These CTCs were able to destroy 4T1/*Ido1−* cells and 4T1/*Ido1+ *cells. However, their cytotoxic effects were higher against 4T1/*Ido1−* cells, most likely due to a higher sensitivity of these cells to apoptotic signaling. (2) In the absence of major immune mechanisms in SCID/beige mice, the growth of *Ido1−* tumors was accelerated, suggesting a role for the immune system in *Ido1*-dependent control of tumor growth. These findings are consistent with previous publications, demonstrating the role of IDO1 in tumor immunosuppression [5–9, 12–14, 37].

Activation of *Ido1* by IFN-*γ* links inflammation and cancer immune surveillance [11, 28, 35], and an increase in IFN-*γ* within the tumor microenvironment also induced IDO1 expression in APCs and DCs that localize to the tumor-draining lymph nodes [38]. We found that IFN-*γ* treatment induced *Ido1 *expression in *Ido1+* cells and had no effect on *Ido1−* cells. Recently, a mouse genetic study revealed a critical role for IDO1 in supporting inflammatory skin carcinogenesis [26]. An analysis of IDO1 dysregulation in cancer showed that JAK/STAT and NF-*κ*B signaling are also essential for IDO1 induction in oncogenically transformed skin epithelial cells [39]. The anti-inflammatory agent, ethyl pyruvate, was found to be able to block *Ido1* expression, indicating a link between IDO1 and inflammatory processes [39].

For the first time, we showed that *Ido1* regulates tumor metastasis via immunosuppressive mechanisms. *Ido1+* tumors develop dramatically higher levels of spontaneous pulmonary metastasis in immunocompetent mice than *Ido1−* tumors. Levels of lung metastasis in SCID/beige mice (deficient in T cells, B cells, and NK cells) are higher than in immunocompetent mice, thus confirming an immunosuppressive role for IDO1 in spontaneous metastasis formation.

In contrast to our expectation, growth of the *Ido1−* tumors was slower than growth of *Ido1+* tumors in immunodeficient SCID/beige mice, suggesting that some *Ido1*-associated nonimmunological mechanisms may be involved in tumor cell growth regulation. It is conceivable that *Ido1− *cells may have an increased sensitivity to apoptosis signaling or slower cell cycle progression and proliferation than *Ido1+ *cells. Our *in vitro *study revealed multiple differences between *Ido1− and Ido1+ *cell populations suggesting the complexity of IDO1- regulated tumor cell functions.* Ido1− *cells grow slower than *Ido1+* cells. Similarly, the inhibition of IDO expression in rat NMU-induced breast cancer showed the reduced cell proliferation *in vitro* [40].

*Ido1+* cells have small proportions of apoptotic cells in normal media and in starved conditions, and they recover better after starvation. In contrast,* 4T1/Ido1−* cells, in which the* Ido1 *gene was down-regulated via shRNA transfection, have dramatically increased sensitivity to apoptosis, thus suggesting an important antiapoptotic function of *Ido1 *in tumor cells. However, molecular pathways involved in *Ido1*-associated apoptosis signaling remain virtually unexplored, offering new areas for investigation.

 Cell cycle distributions within *Ido1− *and* Ido1+ *tumor cell populations also differ in that *Ido1+* cells show a higher proportion of cells in S and M phases than* Ido1− *cells. Using a comparative RT-PCR analysis of cell cycle regulatory gene expression, we found that downregulation of *Ido1* is associated with decreased expression of multiple genes involved in regulation of G1/S transition, S phase, G2/M transition, checkpoints, and M phase, whereas upregulation of *Ido1* induces expression of these genes. Protein analysis of the products of two genes, stratifin (*SFN*) and *ATM*, in *Ido1+* and *Ido1−* cells show that the up- and downregulation of these genes correlates with the increased/decreased production of their respective proteins. Thus, the roles of *Ido1* in breast tumor growth are complex and suggest the involvement of additional nonimmunological mechanisms.

## 5. Conclusion

Our primary finding is that *Ido1* expression has immunological and nonimmunological effects on breast tumor growth and spontaneous pulmonary metastasis formation. We demonstrated that IDO1 is involved in the regulation of tumor cell proliferation *in vitro* and *in vivo*. *Ido1* expression also affects apoptosis in tumor cells. The involvement of IDO1 in cell cycle regulation and tumor cell stress response, in addition to the known role of IDO1 in tumor immunosuppression, make it an attractive target for antitumor therapy.

## Figures and Tables

**Table 1:** Spontaneous lung metastases formation by 4T1/*Ido1+*, 4T1/*Ido1−* or NT-5/*Ido1+*, NT-5/*Ido1−* tumors in immunocompetent and immunodeficient mice.

Mice^1^	No. of spontaneous pulmonary metastases	
	4T1/*Ido1+* tumors	4T1/*Ido1−* tumors
BALB/c	56; (41, 51, 56, 66, 182)	21; (6, 12, 21, 24, 29)*
SCID-beige	54; (23, 24, 54, 75, 164)	69; (33, 54, 64, 69, 69, 71, 77, 92)

Mice^1^	NT-5/*Ido1+* tumors	NT-5/*Ido1−* tumors

FVB/N	6; (0, 2, 3, 5, 6, 7, 8, 11, 14)	1.5; (0, 0, 0, 0, 3, 4, 8, 10)*
SCID-beige	25; (11, 12, 16, 24, 25, 26, 28, 32, 44)*	13; (0, 3, 12, 13, 13, 13, 14, 15)

^1^BALB/c and SCID-beige were inoculated s.c. with 2.5 × 10^4^ 4T1/*Ido1+* or 4T1/*Ido1−* tumor cells. FVB/N and SCID-beige were inoculated s.c. with 5 × 10^6^ NT-5/*Ido1+* or NT-5/*Ido1−* tumor cells. When tumors reached about 2 cm in diameter, lungs were harvested and fixed in the Bounce solution. Metastases were counted under dissecting microscope. *Significantly (*P* < 0.05) differ from all other groups.

**Table 2:** *Ido1 *and the cell cycle genes expression in mouse breast tumor cells.

		Fold changes in cell cycle genes expression	
		4T1/*Ido1+* versus 4T1/*Ido1− *	NT-5/*Ido1−* versus NT-5/*Ido1+ *
		G1 phase and G1/S transition	

1	CAMK2a	1.3	5.4
2	GPR132	4.8	1.8s
3	ITGB1	*− * **17.6**	1.8
4	PPP2r3a	5.1	2.9
5	PPP3ca	*− * **4.2**	2.1
6	SKP2	*− * **3.8**	1.5

		S phase and DNA replication	

7	DNAJC2	7.7	1.5
8	MKI67	8.8	2.3
9	MRE11a	*− * **4.9**	1.2
10	MSH2	*− * **3.3**	1.5
11	PCNA	*− * **5.4**	1.1
12	RAD17	*− * **6.1**	1.8
13	RAD51	*− * **5.2**	1.3

		M phase	

14	BRCA2	*− * **7.1**	1.8
15	CCNA1	19.1	4.4
16	CCNB1	*− * **10.9**	*− * **6.8**
17	CDC25a	*− * **3.1**	1.9
18	NEK2	*− * **4.4**	1.1
19	NPM2	*− * **1.7**	3.1
20	PRM1	10.0	1.6
21	RAD21	3.3	2.8
22	SMC1a	8.6	1.4
23	STAG1	*− * **5.8**	3.0

		G2 phase and G2/M transition	

24	CHEK1	*− * **31.2**	1.3
25	DNAJC2	*− * **7.7**	1.5

		Cell cycle checkpoint; cell cycle arrest	

26	BRCA2	*− * **7.1**	1.8
27	CDK5rap1	*− * **6.5**	1.2
28	CDKN1a(p21)	11.6	2.0
29	CDKN1b(p27)	*− * **6.8**	1.0
30	CDKN2a(p16)	9.9	2.4
31	CASP3	*− * **5.6**	1.4
32	CHEK1	*− * **31.2**	1.3
33	DDIT3	*− * **1.8**	14.3
34	DST	*− * **11.2 **	7.3
35	HUS1	*− * **7.7**	1.0
36	INHA	10.0	5.0
37	MACF1	*− * **8.7**	3.1
38	NOTCH2	*− * **1.8**	3.1
39	PKD1	*− * **2.0**	3.5
40	PMP22	*− * **1.9**	6.1
41	SFN	12.0	*− * **7.0**

		Regulation of the cell cycle	

42	ABL1	*− * **3.4**	1.3
43	ATM	*− * **18.3**	1.7
44	BRCA1	*− * **12.5**	1.3
45	CCNC	*− * **11.0**	1.0
46	PBL1	*− * **8.9**	1.5
47	E2F3	3.3	*− * **1.2**
48	TRP63(p63)	*− * **27.3**	6.8

4T1: stable clone with shRNA-negative control vector pGeneClip/Neomycin; 4T1/*Ido1−*: stable clone with shRNA for IDO.

NT-5: stable clone with control vector pRc/CMV/Neomycin; NT-5/*Ido1+*: stable clone with cDNA IDO.

*A positive value (normal font) indicates fold of gene upregulation; a negative value (bold) indicates fold of gene downregulation. Genes which were found up/downregulated more than 3-fold at least in the one of the cell line are presented.
